# From Curriculum to Consultation: Implementing Epilepsy Education in Primary Care

**DOI:** 10.7759/cureus.110106

**Published:** 2026-06-02

**Authors:** Stjepan Skudar

**Affiliations:** 1 Family Medicine, Zagreb West Health Center, Zagreb, HRV

**Keywords:** antiseizure medication, epilepsy education, family medicine, primary care, referral pathway, shared care

## Abstract

Epilepsy is usually managed by neurologists and epilepsy specialists, yet many practical aspects of long-term epilepsy care occur in primary care. Family physicians frequently encounter patients with epilepsy during consultations for prescription renewals, intercurrent illness, mental health symptoms, contraception, pregnancy planning, injuries, preventive care, and medication interactions. The International League Against Epilepsy (ILAE), a global professional organization dedicated to epilepsy care, education, and research, has recognized the importance of primary care through its Primary Care Curriculum and related educational initiatives. The next challenge is implementation: translating epilepsy education into brief, practical, and repeatable actions within routine family medicine consultations. This editorial argues that primary care epilepsy education should move from curriculum to consultation through checklists, red-flag criteria, urgency stratification, structured review intervals, referral triggers, shared-care templates, clearer communication between neurologists and family physicians, and practical tools that help primary care clinicians bridge gaps in access to neurological care.

## Editorial

Epilepsy is commonly viewed as a condition primarily managed within specialist neurological services. This is understandable, given the complexity of seizure classification, diagnostic evaluation, antiseizure medication selection, and management of drug-resistant epilepsy. However, many clinically important aspects of epilepsy care take place outside the neurology clinic. Patients with epilepsy may consult their family physician for prescription renewals, intercurrent illness, fatigue, mood symptoms, contraception, pregnancy planning, injuries, work-related concerns, preventive care, or questions about drug interactions. Although such encounters may not be labeled as epilepsy visits, they frequently involve issues that influence seizure control, safety, adherence, and quality of life [1]. This issue is increasingly relevant in the context of growing demand for neurological care, aging populations, and limited access to neurologists in many healthcare systems.

The role of primary care in epilepsy has already been recognized by the International League Against Epilepsy (ILAE). The ILAE Primary Care Curriculum provides a structured framework for the knowledge and skills needed by primary healthcare providers caring for people with epilepsy [2]. ILAE educational resources and online learning opportunities further support clinicians who may be the first or most frequent point of contact for patients with epilepsy [3]. These initiatives are important because the treatment gap in epilepsy is not only a matter of diagnosis and medication availability, but also of education, continuity, and practical follow-up.

The next challenge is implementation. Educational curricula and online courses are essential, but their impact depends on whether their key messages enter routine clinical workflows. In other words, the remaining gap may be less a lack of educational content than a "last-mile" problem: how to make epilepsy education visible, usable, and repeatable during ordinary primary care encounters. Family medicine consultations are often brief, problem-oriented, and shaped by competing priorities. A patient may attend for insomnia, anxiety, contraception, a medication refill, or an injury after a fall, while the epilepsy-related significance of these issues remains implicit. Therefore, epilepsy education for primary care should not only define what clinicians need to know, but also support when and how this knowledge can be applied during everyday consultations.

Family physicians are well placed to identify problems that may remain hidden during specialist follow-up. Missed medication doses, adverse effects of antiseizure medications, alcohol use, sleep deprivation, psychosocial stress, depression, anxiety, stigma, and driving concerns may emerge more naturally in primary care. These issues are clinically relevant, yet patients may not always mention them during short neurology appointments, particularly when the focus is on seizure frequency and medication adjustment. Depression and anxiety are especially important, as they are common in people with epilepsy and may affect quality of life, adherence, and outcomes [4].

A practical shared-care approach could strengthen the educational value of existing ILAE resources. Epilepsy-related prompts could be incorporated into prescription renewal visits, chronic disease reviews, reproductive health consultations, and mental health assessments. Family physicians could routinely ask about adherence, adverse effects, seizure recurrence, sleep, alcohol intake, mood symptoms, driving, pregnancy intentions, and new medications. These questions do not replace specialist assessment, but they may identify patients who need counseling, medication review, or timely re-referral to neurology.

Practical implementation should include red-flag criteria, urgency stratification, and structured review intervals. Red flags for prompt neurology review may include new seizure types, increased seizure frequency, seizure-related injury, suspected adverse effects of antiseizure medication, pregnancy or pregnancy planning, uncertainty about diagnosis, or concerns about driving eligibility. Urgency stratification may help distinguish issues requiring urgent specialist input from those suitable for scheduled review, such as stable patients requiring medication adherence assessment or lifestyle counseling. Structured primary care review intervals, for example, during medication renewal or chronic disease follow-up, could help ensure that epilepsy-related concerns are not missed.

In women of childbearing potential, the role of primary care is particularly important. Repeated family medicine visits provide opportunities to discuss contraception, folic acid, pregnancy planning, and the risks of unplanned pregnancy while taking antiseizure medication [5]. These discussions should not be delayed until pregnancy occurs. Primary care is often the setting in which contraception is prescribed, pregnancy intentions are first discussed, or medication safety questions arise. For this reason, reproductive counseling should be a routine component of shared epilepsy care.

Driving regulation counseling also deserves explicit attention. Driving restrictions can have major social, occupational, and psychological consequences for patients with epilepsy, and advice may need to be repeated over time. Family physicians should reinforce locally applicable driving regulations, particularly after seizure recurrence, medication changes, suspected non-adherence, or uncertainty regarding driving eligibility. Because driving regulations differ between countries and may change over time, uncertain or complex cases should prompt communication with the treating neurologist or referral for specialist review.

Neurologists can support implementation by providing concise and practical information in consultation letters. These letters should ideally include the epilepsy diagnosis or seizure type when known, the current antiseizure medication plan, adverse effects to monitor, relevant safety advice, pregnancy-related considerations where applicable, driving recommendations according to local regulations, and clear criteria for re-referral. Standardized neurology-to-primary-care communication templates could make these elements more consistent and easier to apply in routine practice. Electronic health record prompts may also help remind clinicians to review adherence, adverse effects, seizure recurrence, mental health, pregnancy intentions, driving status, and re-referral criteria during relevant primary care encounters.

Table 1 introduces a practical checklist that may help translate epilepsy education into routine primary care [2,3].

**Table 1:** Practical epilepsy-related issues for review in primary care: adapted from ILAE primary care educational resources ILAE, International League Against Epilepsy; EHR, electronic health record

Area for review	Relevance to epilepsy care
Medication adherence and adverse effects	Missed doses and intolerable adverse effects may worsen seizure control.
Sleep, alcohol, and lifestyle triggers	Modifiable factors may lower seizure threshold in susceptible patients.
Mental health screening	Depression and anxiety may reduce quality of life and adherence.
Drug interactions	New prescriptions may interact with antiseizure medications or affect seizure threshold.
Pregnancy and contraception	Reproductive counseling is important before an unplanned pregnancy occurs.
Red flags and re-referral	New seizure types, increased frequency, injury, pregnancy, adverse effects, diagnostic uncertainty, or driving uncertainty may require specialist review.
Urgency stratification	Urgent concerns should be distinguished from stable issues suitable for scheduled follow-up.
Structured review intervals	Medication renewal and chronic disease reviews can be used to reassess epilepsy-related concerns.
Driving counseling	Patients may need repeated advice according to local regulations, especially after seizure recurrence or medication changes.
Communication and EHR prompts	Templates and electronic prompts may support consistent shared-care communication.

This implementation focus does not diminish existing educational work. On the contrary, it builds on the ILAE Primary Care Curriculum and related initiatives by asking how they can be embedded into practice. Brief checklists, standardized neurology-to-primary-care communication templates, referral triggers, electronic health record prompts, and locally adapted continuing medical education modules may help convert curriculum content into patient-level benefit. Such tools should be adaptable to local healthcare systems, because the responsibilities of family physicians, neurologists, nurses, and pharmacists differ across countries.

In conclusion, epilepsy education for primary care should now move from curriculum to consultation. Existing ILAE initiatives provide a strong foundation. The next step is to ensure that their key messages are operationalized in the everyday settings where many patients with epilepsy seek care.
